# The Differences across Future Teachers Regarding Attitudes on Social Responsibility for Sustainable Development

**DOI:** 10.3390/ijerph17155323

**Published:** 2020-07-24

**Authors:** Ligia Isabel Estrada-Vidal, María del Carmen Olmos-Gómez, Rafael López-Cordero, Francisca Ruiz-Garzón

**Affiliations:** 1 Department of Research Methods and Diagnosis in Education, Faculty of Education and Sport Science, University of Granada, 52071 Melilla, Spain; ligia@ugr.es (L.I.E.-V.); fruizg@ugr.es (F.R.-G.); 2 Department of Didactics of Social Science, Faculty of Education and Sport Science, University of Granada, 52071 Melilla, Spain; raloco@ugr.es

**Keywords:** education for sustainable development, community-university partnership, social responsibility, sustainable development, environmental justice, community-engaged research

## Abstract

In the search for sustainable development, in which the ecological footprint is carefully considered by consumers and companies, teachers play an important role within a social and economic framework. This role relates to aspects of social responsibility. It should involve knowledge about education for responsible consumption in order to care for the environment both individually and socially. Considering this, the aim of this study is to find out whether there are differences in the level of awareness and the habits of future teachers of Early Childhood and Primary Education regarding sustainable social responsibility. A non-probabilistic sample of 30 Early Childhood Education degree students and 22 Primary Education degree students was used. Semi-structured interviews and an inductive process were conducted to examine the importance of Sustainable Development in society, the relevance of Social Responsibility for Sustainable Development (individual versus corporate), the attitudes and habits relative to Sustainable Development and the education on Sustainable Development in schools: knowledge, attitudes, and proposals. Students agree that they consume excessively. This is everyone’s individual responsibility (as regarded by all participants), although changes could be supported by institutions and companies (Early Childhood education students argue in favour of corporate responsibility). Knowledge deficits were identified in relation to production, distribution, and sale processes. They consider education to be the main factor for sustainability, while society is ranked as the least important, observing an evident disagreement in relation to environmental and economic factors (perception of collective responsibility; Early Childhood versus Primary Education students). Finally, they also outline teaching proposals (active and participatory) to foster education for sustainable development at schools.

## 1. Introduction

People’s awareness and behaviour may favour the environment as a result of their Social Responsibility for Sustainable Development (SRSD), thus improving environmental justice [1,2]. Through SRSD, future teachers may be able to improve environmental, social, and economic conditions and therefore enhance protection against environmental degradation by their daily actions or by educating. They may act both as individuals and as education professionals with the capacity to influence others, to a greater or lesser degree, and so they play an interesting role as committed citizens within the university community.

Their contribution is related to the type of SRSD they hold, the development factors that are most relevant in a country, as well as to the various aspects included in the Sustainable Development Goals (SDGs). Moreover, it is interesting to ascertain whether future teachers specialising in Early Childhood Education or Primary Education show differences regarding their level of awareness and habits as part of the development of SRSD.

There are multiple interpretations of the perceptions of Sustainable Development (SD) and the Sustainable Development Goals (SDGs), which are ultimately aimed at satisfying the needs of both current and future generations [3,4,5]. The reference document that facilitates the implementation of the SDGs through a series of guidelines with global applicability is the action plan known as Agenda 2030, which builds on the Millennium Declaration of 2000 and is the tool currently used in addressing the objectives of SD [3,6]. Social and individual well-being, in addition to the values of human rights, justice, or health, are aspects that are circumscribed therein [3,7], fully respecting the interrelationship between environmental development, economic development, and social development [4].

The intention is that by 2030, the population will be informed and aware of SD and lifestyles in harmony with nature [3]. Since SD and human nature go hand in hand, the former could be based on ethics [3,8]. Thus, people at an individual level can be responsible agents of SD through the lifestyle they choose to adopt. As a way of example, choosing certain types of tourism and food consumption, purchasing items with the cheapest prices or fashion trends (such as new technologies or clothing items), taking care of objects already possessed to increase their longevity, selecting products that generate less waste, and recycling or implementing energy consumption habits are actions that can be targeted in daily life to take care of the environment individually and be socially responsible for it [7,9,10,11,12,13,14]

From an economic development perspective, a consumer with Individual Social Responsibility for Sustainable Development (ISRSD) may be able to condition the economic model of companies, since the production and sale of products vary according to the social demand [15,16,17,18]. Likewise, an individual may also act to promote Corporate Social Responsibility for Sustainable Development (CSRSD) as a member of a company, which could contribute to the well-being of society [19]. Their responsibility as agents of pro-environmental social change is centred on decision making and the implementation of economic actions, which are mainly influenced by the most sustainable ones, such as a common good economy, fair trade, or circular economy, with the capacity to address products to an ethical consumer, to a greater or lesser extent [17,20,21,22,23,24]; all this is based on the creation of a European ethical market [25]. Therefore, companies would be able to offer more sustainable products, and as a result, favour specific consumption trends [13,14,17,26].

In the same way, an individual may engage in CSRSD as part of public administrations and other entities of a public or private nature, such as public organisations with legislative or educational competence, non-governmental organisatiosn (NGOs), environmental cooperatives, etc. [27]. In the field of education, people may intervene as a social group to promote management and/or Education for Sustainable Development (ESD), given that social norms shape people’s behaviour [28]. For instance, public institutions may be able to promote the social development of environmentally friendly competencies and actions through educational policies [29,30] within the context of government policies [31,32].

However, one can also participate as an involved and responsible teacher of ESD acting with CSRSD. Some examples are the inclusion of sustainability as cross-curricular content, specific educational programmes on sustainability that are implemented throughout school, and training courses for teaching staff with the aim of knowing how to intervene with students, the educational community, and the local setting in which the school is found [33,34,35]. At the same time, the degree of Sustainable Social Responsibility for Development (SRSD) of a teacher as an individual is essential, since their SD behaviour could be an example not only for students but also as a responsible citizen (person with ISRSD).

For this reason, schools approach ESD from early ages [36,37]. This may include projects such as Eco-schools [38,39] or Young Reporters, which favour the development of sustainable competencies in those who will later become university students. An example of this is that they are promoting sustainable foodstuffs from both conventional and alternative agriculture, through which they can educate the population via healthy diets and school gardens/vegetable plots [40,41]. However, schools’ management has to deal with the availability of resources and their use by the educational community. This entails the improvement of facilities, reduced energy and water consumption, reduced waste [42,43,44], care of school furniture, better decision making, the purchase of information and communication technology (ICT), etc.

All of this implies the presence of dynamics that enhance the active participation of individuals at the education centre and promote the implementation of innovative methods that encourage learning how to identify problems and solve them [45]. These dynamics increase the knowledge of environmental problems generated by irresponsible lifestyles, and they also help implement assessment methods that enable teachers to determine the participants’ level of learning. Furthermore, issues could be worked holistically [46] by linking content from diverse educational topics, such as responsible sustainable consumption and sustainable health [47].

To achieve this, teaching staff should be made aware of their responsibility to society [48] as professionals with capacity to influence the competencies of their students (students with SRSD). This should be crucial in their training so that their students are taught to act pro-environmentally on a daily basis within their individual environment [18,45]. Thus, future teachers must increase their awareness in order for them to act as individuals with increasingly more responsible consumption habits [49]. These habits may foster improvements within their environment, and their lived experiences may also broaden their understanding of the difficulties of educating environmentally conscious students (students with ISRSD).

Although in-service teachers present adequate levels of environmental knowledge and attitudes, they lack environmental action strategies [50]. The significance of this group of professionals is to be found in their capacity for improving the quality of education (SDG 4). Moreover, ESD is specified within SDG 4.7 [51]. Accordingly, it is considered that this population group should be further studied.

In the case of Higher Education (HE), it can be said that it is a source of learning about more pro-environmental private and professional lifestyles for students. For that reason, this level of education can act as a driving force in the acquisition of knowledge, attitudes, habits, and ultimately, competencies that help develop sustainability in our daily lives as a tool to improve and preserve the environment [10,18,45,46,52,53,54]. Planning and implementation, empathy, compassion, and solidarity, among others, are highlighted as key competences favouring SRSD [55]. It must be kept in mind that those competencies associated with the construction of inter-personal, strategic, and normative norms are better attained by students of higher education [56].

These competencies can be developed through the university guidelines on SD, which aim at ensuring that all graduates acquire basic competencies that help them integrate a sustainability perspective in their professional and personal activities by incorporating SD in their syllabus [57,58,59]. Currently, there are syllabuses where ESD is being established as a specific course, not only in degrees with competence in the field of educational sciences, but also in other degrees in the field of experimental sciences [60]. In other cases, a greening of the curriculum carried out by some professors is also found [61,62], which deepens the knowledge acquired in previous stages, since HE should not only provide specific models of excellence in education, but also guidance to the community on social improvement and environmental sustainability in order to develop the proper competencies [18,48,56,63,64,65,66].

In the case of professors who lecture on Early Childhood and Primary Education degree programmes, they believe that it is possible to include contents and approaches on sustainability in the courses they teach or coordinate. However, although they state that they integrate the values of respect, equity, and tolerance as aspects of SD, they do not do the same with other SD values. Reasons include the lack of awareness of the problem, lack of involvement, and lack of training [67]. Thus, among the actions that favour SRSD, they have sought to raise awareness of the impact of human actions on the environment or on local and regional systems [68], the use of service learning methodology in NGOs [51], or the development of social work based on ecological and environmental justice within SD [69].

This being said, reality shows that there is still a long way to go and that ESD could be implemented more extensively in university studies [29,45,53,56,62,63,64,66]. This is highly relevant in the case of teachers who are training to be professionals who will practice in any society where education is compulsory. That is to say, it is critical that they are made aware of the need to intervene at school and know how to educate (person with CSRSD), modelling a lifestyle that is in line with pro-environmental values (person with ISRSD) [45,62]. As a consequence, HE professors should ask themselves whether universities are training students to face the global challenges of society with responsibility [70,71], especially regarding aspects such as the problems of social sustainability and social and environmental injustice. This is a pending issue in HE institutions [72], whose syllabuses should include more critical aspects [73].

That is why there are research studies that focus on the creation of instruments for Social Responsibility (SR) aimed at helping universities to modify their curriculum [74], or on the differences among students. For instance, it has been found (1) that university students have a greater concern for the social and environmental dimensions of sustainability (versus the economic one) [75], (2) that the female gender has a greater tendency toward ethics, Social Responsibility (SR), and SD (within the concept of SRSD) [76], and (3) that University Social Responsibility (USR) is still incipient compared to SRSD [77].

Given what has been presented above, society is responsible for taking care of the environment at both the individual and social level [52], having the task of acting on their environment, taking care of it, and resolving problematic situations [46]. It is here where teachers have to be responsible for their actions, both as individuals and as citizens who develop competencies for environmental respect [78] and bring about local changes with a global influence (SRSD), such as within the public administration (CSRSD). Their function is to act as agents within educational administrations and influence the society within which students operate [54].

For this reason, the objective of this study is to find out whether there are differences in the level of awareness and the habits of Early Childhood and Primary Education teachers in training regarding sustainable social responsibility.

## 2. Methods

### 2.1. Participants

The sampling used was theoretical or intentional, and it was based on the information needs identified in this study and on the level of training of the participants, so only the final year students (4th academic year) of the two degrees of teachers in training were consulted. The size of the sample coincides with the total number of students of that academic year: n = 30 students enrolled in an Early Childhood Education degree (57.7%) and n = 22 students enrolled in a Primary Education degree (42.3%). This provided a total of 52 future teachers from Andalusia (Spain). Participants had an average age of 20 years (SD = 3.837). Out of these, 7.5% were male (n = 4) and 92.5% were female (n = 49).

All subjects gave their informed consent for inclusion before participating in the study. The study was conducted in accordance with the Declaration of Helsinki, and the protocol was approved by the Vice-deanship for Practices, cooperation and social responsibility of the Faculty of Education and Sports Sciences of the University of Granada Ethics Committee (Code ML.04-11-19).

In order to ensure the scientific rigour of the study, the four criteria pointed out by Olabuénaga [79] for qualitative studies have been followed. To begin with, the credibility criterion has been ensured through the triangulation of the information sources and the saturation of the data, as well as through the consensus and supervision of the reports by the informants. Then, the transferability has been addressed by interviewing all the students who make up the 4th academic year of the different programmes on the campus, who were undertaking an internship at the time of conducting the research. Finally, the dependency and confirmability criteria have been validated by external auditors who have supervised the decisions made in the collection and interpretation of the data in order to detect bias or private interests, thus avoiding spurious elements in the study.

### 2.2. Instrument

Semi-structured interviews were used (see Table 1).

In the present research study, an initial ad hoc interview was prepared that was based on and adapted from that developed by Olmos et al. [62]. Content validity was determined following administration of the instrument to a sample with similar characteristics. In order to carry out the analysis (expert panel), 14 experts participated whose responses served to evaluate the content validity index. The expert panel was requested to rate the clarity, coherence, relevance, and sufficiency of each interview item designed to measure future teachers’ opinions about the importance of sustainability and the development of sustainability habits. This was carried out by reporting agreement on a four-point Likert scale (1 = inadequate, 2 = barely adequate, 3 = somewhat adequate, and 4 = highly adequate). Elements were only considered if they obtained response percentages from the expert panel that were (3) somewhat adequate or (4) highly adequate. All items obtaining a value lower than 89% were eliminated. Following this, interview coherence was examined, showing excellent preliminary content validity.

Once the interview with the future teachers was completed, content validity was again assessed using the same procedure. In this case, results obtained from semi-structured interviews with future Early Childhood Education and Primary Education teachers in the 2019–2020 academic year showed a value of 94%. This provides evidence of a correct and adequate content validity.

### 2.3. Process

#### 2.3.1. Data Collection

Once the protocol of the ethics committee of the University where this research was carried out approved the study, and following the ethical recommendations established by the Declaration of Helsinki, anonymity and consent to participation were guaranteed. Semi-structured interviews were conducted with future Early Childhood Education teachers and Primary Education local teachers during the 2019–2020 academic year. The interviews lasted from 20 to 35 min. An initial explanation of the study was provided, and participants were offered the opportunity to clarify any doubts about sustainability.

#### 2.3.2. Data Analysis

Interviews were analysed through ESD thematic analysis. Data were included using an inductive process, and responses were evaluated in agreement with the data obtained. This provides new perspectives from which reality can be better understood [80,81]. The specific process involved a manual transcription of interviews, followed by the generation of initial codes that were applied to the data. These codes were extracted through the analysis of the responses given to the 14 questions posed to future teachers during their semi-structured interviews.

The first 4 questions provided demographic data. The code describing the importance of responsible consumption was obtained through analysis of questions 5, 6, and 7. Agents relating to responsible consumption were coded through analysis of questions 8, 9, and 10. The codes obtained for the development of individual attitudes toward the environment came from question 11. Analysis of question 12 provided the code describing students’ habits toward environmental care as individuals. Finally, the level of knowledge about education for responsible consumption was measured in questions 13 and 14 (see Appendix A Table A1).

Subsequently, the responses pertinent to each code were grouped in accordance with common content, and finally, sub-codes were identified (see Table 1).

The entire interview process was analysed in Spanish; however, interviews were later translated into English by a professional translator. Data analysis was performed using the latest version of the qualitative software NVivo (International Qualitative Software Research (QSR International), Melbourne, Australia, 2020).

The reliability analysis of the data was performed in consideration of the agreement between coders with regard to the codes and sub-codes created by three researchers. This was done through agreement and concordance, via data triangulation. Outcomes ranged from K ≥ 72 to K ≥ 96. The different codes and sub-codes were revised and modified before being included in the analysis.

Finally, a fourth external researcher who was not familiar with the research analysed 70% of the interviews. After the analysis performed by this researcher, the codes and sub-codes resulting from it were compared with the rest. This yielded a concordance correlation of ≥ 86.

## 3. Results

### 3.1. Thematic Analysis of the Responses Given by Future Teachers

Once the data analysis was conducted, four main themes for the study of opinions about ESD in future teachers were revealed: (1) Importance of SD in society, (2) Relevance of ISRSD versus Corporate Agents, (3) Attitudes and habits relative to SD, and (4) Education for SD in schools: knowledge, attitudes, and proposals.

These themes were also divided into sub-themes. The categorisation system used in this thematic analysis is shown in Table 1 and Appendix A Table A2.

#### 3.1.1. Importance Attributed to Sustainable Development (SD) in Society: SD as an Important Issue in Society and a Factor of Progress in a Country

No differences existed between Early Childhood Education students (ECES) and Primary Education students (PES), with responses unanimously indicating that the current consumption of individuals is excessive, and thus, it is an environmental issue that should be tackled by society. In general, the consumption of energy resources, water, and transport stand out, both at the society level and in schools.

“Parents bring their children to school in cars, even though their houses are close. Classrooms lights and computers are left switched on in empty classrooms.”

(ECES9_SD)

“Yes, nowadays people have more than what they really need.”

(PES10_SD)

“Consumption can be considered to be excessive, especially in our city (…), consumption of packaged foods is promoted a lot or the excessive use of cars.”

(PES17_SD)

In relation to country development, a large diversity of responses exists among ECES and PES groups. Certain factors are considered for determining country development. These include education, environmental development, economic/working environment, and social aspects. All participants considered education to be the most relevant factor and social aspects to be least relevant. The ECES group had a greater tendency to consider economic–work factors as the second most important, followed by environmental factors, which was in the third position. The PES group reported the opposite order of importance. The ECES group seemed to have a deeper understanding of aspects related to the economy, given that they consider it to be the basis of all other factors. Within this conception, education is a vehicle for training and raising awareness.

“For a country to consider itself developed, it should focus on everything globally, as one leads to the other.”

(ECES31_FDP2)

“Educational, because training individuals is important to have competent people at work. Economic, as the economy enables education and training; it allows projects to be carried out in all fields (educational, health, engineering…); it creates jobs…”

(ECES28_FDP1)

“The educational context; if there is education, the rest will fall into place. It is the dominant factor. The environmental [context]; if we don’t take care of our planet, there will be no planet, [and] then the rest is useless. For this, there must be prior education (on sustainability).”

(PES1_FDP2)

“All of the factors are important for a country to be considered developed, in the following order: environmental, educational, social, economic (…) because through education, we can incorporate the recommendations that are necessary for all of us to be able to contribute towards making a sustainable and developed world (…).”

(PES20_FDP37)

#### 3.1.2. Agents of SD: Social and Educational

Students of both groups ECES and PES believe that as social agents, all individuals are personally responsible for collaborating to make the world more sustainable. This does not mean that public and private bodies are relieved from the responsibility of developing society, recognising the importance of politicians, companies, and administrations to this effort. They consider that all of them should join forces and work together to pull in the same direction. In the case of individual involvement, each person can raise awareness in others by appropriately sorting household waste, using recyclable bags, and using more economical means of transport such as bicycles or walking. In the case of public and private entities, actions should be directed towards raising awareness, training, and complying with actions regarding SD.

“Everyone (…); as individuals, it is us who create businesses and public administrations. Thus, it is the job of each and every one of the people who live on the planet to make it a sustainable place, both individually, in our personal and daily life, and collectively, trying to raise awareness in others, (…).”

(ECES3_2A)

“We are all responsible and [it is] necessary to make a more sustainable world, although the administration is the one that can manage to make it easier and more effective through laws.”

(ECES15_2A)

“All people (…) recycling every day, saving water and energy, doing the shopping with bags made of recyclable materials, avoiding the use of plastic, and throwing rubbish away in the right places (…) to change the planet for the better. Public administrations must be advised and trained on sustainability to then train citizens.”

(ECES10_2A)

“Making a more sustainable world is the responsibility of all inhabitants of the planet. (…) This task requires collaboration and work from all public administrations and companies, as well as individuals and other groups.”

(PES11_2A)

“All together, because there is no point in public administrations doing a good job if individuals are not collaborating or doing the opposite”

(PES12_2A)

There is disagreement between ECES and PES about the importance given to each one of the social agents. ECES assigned greater responsibility to public administrations and institutions, as well as businesses, as the agents with the most repercussions for society. Social acts can be legislated and are directed towards people or companies. They can seek to raise awareness and inform about environmental care, and they can reward pro-environmental actions or decision making that improves business infrastructure in order to create more environmentally friendly products. In another sense, they indicate that the efforts of individuals do not have as much impact when they are ultimately dissipated by the decisions and actions made by social entities, such as for example via inadequate waste management. In contrast, those in the PES group assign greater responsibility to people as individuals. This is because, in addition to having a collective impact, the sum of individual actions is also a part of social entities and, as a result, it can generate change within them.

“It is the responsibility of everybody, but if I have to pick one, then it would be, without a doubt, companies (…), they should change all of their polluting practices.”

(ECES9_2A)

“Public administrations, well, it is these bodies that have the greatest responsibility as they carry out their function.”

(ECES14_2A)

“In theory, the three (public administrations, companies, and individuals) should compete, but in practice, it must be individuals, given that their preferences will condition companies and administrations.”

(PES5_2A)

“A company cannot be held responsible to be sustainable if afterwards you, as an individual, are not willing to do your bit.”

(PES19_A2)

In the case of education in schools, participants consider collaboration and involvement to be relevant at the time of enacting their educational function in school centres as ***educational agents***. In this regard, they consider that education could take place from a very early age in order to develop positive habits and attitudes towards sustainable development. This should mainly occur through programmes and projects that are undertaken throughout formal schooling and not just in an opportunistic manner.

“Sustainability is the responsibility of everyone. Thus, we should teach people from the earliest age about this topic.”

(ECES2_2B)

“(…) to work in all countries through projects and programmes in order to educate about values, (…) respect for living beings and ecosystems and the damage we are doing.”

(ECES20_2B)

“Integrate education for sustainability in culture and school life is not just another project. It is one of the greatest challenges to the construction of sustainable communities, a new way of thinking, a shared focus on development of the school and its improvement.”

(PES_2B)

#### 3.1.3. Attitude and Habits of Students about SD

In relation to the pro-environmental attitudes of participants, differences were not found to exist. Students are aware of the importance of caring for the environment and implementing small changes in daily life to resolve current environmental problems, it being accepted that the sum of individual actions has a large impact. Furthermore, participants consider that it is their duty to take responsibility for this, and one of the tools is information. They are aware that not only does their behaviour have repercussions for the environment (droughts, deforestation, climate change, contamination, and loss of biodiversity), but it also impacts upon the health of people themselves. With regard to the actions that they believe to be most relevant, they mention waste management (especially related with plastic, e.g., recycling or the consumption of products that are not packaged), energy and water consumption (quantity and quality, such as wind and hydraulic energy), and more environmentally friendly means of transport (use of alternative energies, public transport, or bicycle).

“(…) better understand problems of the environment to be more aware that each small wrong gesture performed by human beings damages [the] Earth. (…) such as deforestation, drought, damage to our health, climate change, pollution of the sea, risk of species extinction.”

(ECES19_3A)

“(…) each and every one of us must join in to solve environmental problems as serious as climate change through simple and everyday acts, such as recycling, turning off lights when they are not needed, avoiding the use of plastics as much as possible, etc.”

(ECES27_3A)

“We currently have many environmental problems: pollution, deforestation, climate change, (…). We must all collaborate in daily actions in order to improve the planet. Recycling, using public transport or bicycles for transport, using recyclable packaging, avoiding the use of chemical products, taking care of water, caring for flora and fauna.”

(PES7_3A)

“I always try to do the best possible and, of course, strengthen good habits, promote respect and consideration and responsible consumption.”

(PES22_3AP3)

In relation to the habits of the interviewed students, the majority recognise that they do not engage in responsible consumption or that they just try to, with only six participants from each student group claiming that they consume responsibly. Amongst the habits reported to justify this position, students stated that they did not inform themselves about the manufacturing, transport, and sale processes of the products they consume. Moreover, differences are observed amongst students given that a greater number of ECES indicated that they did not engage in responsible consumption habits (12 out of 30), compared to students in the Primary Education group (4 out of 22). In the case of those who indicated attempting to consume responsibly, respondents attributed their answers to them being aware that their habits can be improved, despite many of them already being pro-environmental (11 PES and 8 ECES).

“We actually consume more than we need, sometimes unnecessary things (…) when we go to the supermarket, we do not stop to look, nor do we even think about the environmental impact of the products we are picking up (…) the manufacturing process, or transport, or distribution (…) the waste this product will leave behind or the time it will take to eliminate it (…).”

(ECES31_3AN)

“I consider that I do engage in responsible consumption. I try to go to places on foot, nothing is left on at home, reusable bags, eat less meat…”

(ECES19_3AP)

Amongst the most pro-environmental habits, students reported a reduction in the consumption of animal products (especially in the PES group relative to the ECES group) and in energy consumption at home, as well as increased mobility by walking and waste management (use of reusable bags and recycling). Moreover, students recognised engaging in better habits in relation to themes that are not related to food consumption. Nevertheless, they argue that shops offer a broad range of packaged and processed products in such a way that it makes it difficult to develop acceptable habits, as can be done with water consumption. Likewise, the media and the intense publicity surrounding people persuade them to make unnecessary purchases.

“With regards to aspects oriented towards food, my consumption doesn’t tend to be as responsible as with other aspects (saving energy, water, recycling); as I am trying to follow a balanced diet, I don’t consider too much the materials in which they are wrapped up in (such as plastic wrapping, bags, etc.).”

(PES2_AP)

“Due to the fact that I like to follow a healthy diet and lifestyle, my consumption patterns mostly revolve around certain food items, avoiding meat, as this leads to [the] terrible mistreatment of animals.”

(PES7_AP2)

“Engaging in sustainable consumption is difficult, as many products are packaged and generate a lot of plastic (lettuce wrapped in plastic, bottled water, and the same for everything).”

(ECES12_AN2)

“(…) the media bombards us with a lot of publicity (…).”

(ECES23_AN2)

#### 3.1.4. Education for SD in Schools: Knowledge, Attitudes, and Approaches

A great deal of information exits about sustainable development amongst university students, with no significant differences being found according to university degree, although six participants indicated having little knowledge and another two reported a greater level of enlightenment. In general, this concept is associated with the responsible consumption of healthy food, energy, and waste, in order to reduce the use of products, resources, and services derived from it. Whilst it is true that many students have not acquired relevant knowledge during their studies within the educational system, others have received such knowledge within the same system, especially at university, through conferences, seminars, and workshops. Nonetheless, a great need exists to provide training through informal education, where the influence of the media and social networks is essential.

“[I know] little, given that I have never received any training about this at school, but from today onwards, I would like to search for information on the topic, options to improve, to change, etc.”

(ECES15_C)

“I know the basics, thanks to all the talks and seminars I have attended during my academic years at primary school, secondary school, and university.”

(ECES17_C)

“[I know] basic aspects such as recycling, saving energy, pollution or the accumulation of litter in the sea and on the land.”

(PES16_C)

Moreover, all respondents considered education about sustainable development to be relevant throughout the entire educational system, starting at early ages both in the classroom and at home. They consider education to be a key factor for families to be able to be role models for students, as well as for teachers at academic centres, as all those responsible for consumption engage in one of them. It was even proposed that a specific subject should be implemented to tackle sustainability at education centres. It should be directed towards students and strive to engage families, either through an extra-curricular subject or a course delivered in the classroom. There is also a small group that considers education as a service model and suggests making use of local facilities (such as parks or ecological associations), in the same way as with the resources available at the school centre.

“From classrooms, we can make children more aware, but the reality is that we must raise awareness in parents so that their children get used to responsibly consuming certain products from early ages and converting this into an everyday activity.”

(ECES8_PESC)

“We should promote it especially in classrooms, as this is where most consumption takes place. Neither teachers nor students are aware of their consumption patterns. For example, when they are allowed to take a break on the playground, change classrooms, or engage in unusual activities, they don’t normally turn off the lights or the computer in the classroom. This is very common in schools.”

(ECES11_PESC)

“(…) through essays, games, workshops, and extra-curricular activities, by means of environmental associations. They should work a lot in nature parks and with Guelaya [local association of ‘ecologists in action’].”

(PES6_PESC)

To this end, routines can be created that conform with working principles that are collaborative, motivational, creative, reflexive, developed from students interests and daily life, active, participatory, and experimental. The type of activities to be incorporated will be games, songs, workshops, videos, the delegation of responsibilities on a daily basis at the academic centre, and/or projects. The only difference between students was that those in the ECES group also mentioned organising debates during daily assemblies, whilst those in the PES group approached consumption in a cross-curricular way through content. Amongst the reasons for this, we can consider the desire to raise awareness of oneself and the environment (social and natural) and value the natural resources to which we have access, to learn about norms and the importance and consequences of irresponsible consumption (production processes, transport, and distribution), and to develop feelings of respect and solidarity. This goes along with efforts to learn how to act in order to reduce the impact of habits or with campaigns targeting unnecessary consumption (such as Christmas promotional campaigns promoting overconsumption). One of the aspects most strongly related to responsible consumption is altruism or selfishness, in the case of seeking health improvement, for instance. In this regard, these aspects will influence knowledge about the impact of behaviours on the economy of their immediate environment (reduction of family expenses and those of the academic centre) and personal health (contaminated foods or diseases).

“Mentioning every morning in assembly the norms for taking care of the environment, which are also found written on cards in the classroom, and for all students to see. For example, choosing the least contaminating means of transport to go shopping, reusing materials, managing water consumption…”

(ECES18_EPC2)

“Responsible consumption, I would strengthen it in a clear and dynamic way. For example, each day, I would put a child in charge of turning off the lights when we leave the room, or turning off the lights if they see daylight coming in. Separate [waste] in classroom using the blue, green, and yellow boxes.”

(ECES20_EPC3)

“Motivating students with games–activities–workshops, allowing them to take the lead (in order to favour their interests and participation with others), using themes related with their daily lives (to see the repercussions and feel more linked to reality).”

(PES1_EPC3)

“(…) all classrooms would have bins for each material (recycling containers: a tub for batteries, used oil…). When explaining the agenda, when the opportunity arises, alternate with something related to responsible consumption, give workshops on this, explain to them risks that exist if we don’t consume responsibly and that they themselves must search for information (…).”

(PES4_EPC2)

Agendas are focused on waste (such as separating or substituting products for others that are less damaging to the environment), water (daily situations such as washing hands or cleaning their teeth), transport (public transport use or car sharing), sustainable health (developing nutrition or taking healthier food that generates less waste to the academic centre), and purchasing objects (toys or foods).

“I would use the moments during assembly to raise awareness in the kids about the importance of responsible consumption, conducting debates. During these, we would talk about when they buy a product and whether they think they really need it, whether they recycle or reuse things, whether they waste water or leave lights turned on for no reason, whether they come walking or by the car… Through this, I would make conclusions about student opinions to work with them in a sustainable way.”

(ECES25_EPC3)

“(…) close to Christmas time. Both fathers and mothers who are excessive consumers and kids (…) through a talk for parents with the help of a professional who would deliver it. And then with the kids to ask them (…).”

(ECES28_EPC4)

“I would motivate them to get around using bicycles or walking, take care of the flora and fauna, avoid using plastic bags, use recyclable packaging, save energy, manage water as it is very important for our planet, produce less waste or reuse paper.”

(PES9_EPC5)

“They would watch a video about the problems in our planet to raise their awareness on responsible consumption. We would start in the classroom and then continue at break-time, leading students to acquire routines and go incorporating it progressively into their lives.”

(PES18_EPC3)

## 4. Discussion

The vision that future teachers have about sustainable social responsibility for the creation of a more sustainable world is different. Future teachers of Early Childhood Education assign greater importance to the administration and public institutions as agents that influence individual actions. They believe that these agents should be trained about sustainability in order to be able to educate citizens and raise awareness. However, they consider teachers as responsible for the advancement of SD. Therefore, future teachers of Early Childhood Education attribute greater importance to sustainable corporate social responsibility by assigning the greatest responsibility not to the individuals within the entities, but to the external locus of control subjects—that is, to educational and environmental policy makers, with the exception of the case in which they include themselves as part of the public administration in educational matters. Conversely, future teachers of Primary Education consider themselves mainly responsible for the actions in the field of SD, thus having a greater sustainable social responsibility, either as individual sustainable social responsibility or sustainable corporate social responsibility, which is found within the various existing organisations. They maintain that individuals are the ones with the greatest responsibility, since their action may be able to modify public institutions, as they are part of the companies and the administrations.

Teachers in training seem to be aware of their responsibility in the educational field as contributors to the quality of education. Thus, among the factors that are beneficial to the development of a country, although there are differences between future teachers of Early Childhood and Primary Education, education is widely recognised as the most relevant one (SDG 4, quality of education) [82].

In general, as it is proposed in the SDG referred to as production and responsible consumption (SDG 12) [7], students agree that excessive consumption exists in society, with responsible consumption (RC) being an environmental problem that could be tackled with better education. To this end, they consider it to be fundamental that they act in an individual way as those responsible for the aforementioned actions, and thus, they would take care of their environment through small daily actions [7,9,10,11,12,13]. This being said, the responsibility of social groups cannot be diminished, as it is crucial that all parties work together collaboratively towards the same goal [14,15].

Nevertheless, PES are intrinsically motivated to take individual responsibility as part of a group, given the influence that they can have as a member of public administrations, companies, and private or public entities. In contrast, ECES attribute greater responsibility to social groups since they favour certain attitudes and behaviours in people at an individual level through legislation, norms, product supply, production, and advertising (extrinsic motivation). This has also been examined in previous research [13,14,22,83].

In case we, as individuals, should make our personal contribution as those responsible for the environment, we could be supported by institutions and companies through pro-environmental changes to the practices of the aforementioned social groups, which will, at the same time, facilitate the functioning of society in an individual way [14,23,26,83]. As already indicated by other authors, norms developed through environmental and educational policies favour environmentally friendly skills and actions in each and every one of us [24,25].

In relation to the personal implication of university students, awareness of the importance of taking care of the environment is evident. Students even consider it to be their duty to act individually in order to achieve global outcomes, with information being the greatest tool available. Students explain that their behaviour affects not only the natural and urban environment, but also their own health. The main reported actions are the management of waste, water, and energy together with mobility. Students identify knowledge deficiencies with regard to the entire production and manufacturing process, transport, and sales of regularly consumed products. These knowledge gaps leave them unaware of the real impact of consumption, not knowing how to act. This argument is corroborated by the fact that they perceive themselves to be influenced by the wide range of packaged and processed goods offered in shops, in addition to advertising in the media and the information shared within social networks.

Concerning their habits, better SD patterns were reported by students in the PES group. In general, students recognise that they do not engage in RC or that they only ‘at least try to engage’ in RC, with a small group indicating that they do engage in RC. The most common habits found included reducing animal-based foodstuffs (especially PES), walking, using reusable bags, separating waste, and improving energy consumption at home. Due to this, an educational challenge in the university environment will be to understand the concept of sustainability as a pillar upon which various aspects can be built upon, such as fair trade or school gardens [41].

Therefore, of all the four factors proposed to be relevant for country development and to show a degree of response variability, education was considered to be the main one, while society was the least explanatory factor. Nevertheless, discrepancies were found with regard to the fact that ECES considered the economic factors to be more relevant than the environmental factors, with many responses being equivalent to those given for education. This can be supported by the fact that a certain economic level is necessary in order to favour the other factors and fulfil the basic needs of individuals. It could be said that these students have a greater perception of the responsibility of groups, attributing them with greater power and resource management capacity, alongside greater social influence.

Notwithstanding, teachers recognise their responsibility as educators in society. They acknowledge that they should seek to intervene with students at their school from an early age. This should reflect not only what they are currently doing at school [37,38], but it should also engage relatives and occur throughout the entire schooling process, without limiting it to particular actions performed at specific occasions. They consider that both teaching staff and families should engage in adequate behaviours with regard to consumption, given that they are role models for students; thus, interventions with families are also required.

According to participants, the teaching methodology to be followed should be active and participatory. This method should use strategies placing an emphasis on collaboration, reflection, information, the assimilation of respectful behaviours, assembly use, and the delegation of responsibilities within academic centres. However, the methodology applied should always focus on topics that are well-understood by the teacher and based on the attitudes and habits that the teacher wants to develop.

## 5. Conclusions

Sustainability is a concept that entails a wide spectrum of social and natural actions, and as a result, it should be understood in this way. Nevertheless, university students reiterate certain themes and contents (waste, mobility, biodiversity, positive health, energy, and water). There are some exceptions in PES, who have a more holistic vision founded on the relationship between the education of responsible consumers and other educational contents. Nonetheless, in general, they provide a stronger vision of sustainability through information and activities that facilitate sustainable development, as both citizens who belong to a society and students.

Thus, teachers engaged in training provision, generally, as a collective, present an appropriate attitude towards SRSD, SD, and education surrounding these topics, despite presenting certain training and habit deficits, which can be addressed through the teaching-focused methodology proposed. In relation to this, it is clear that the educational context has an increasingly greater impact on individuals engaging in more actions in formal, informal, and non-formal contexts. University students still require educational interventions that enable them to act as those responsible for SD [24,56,64,66]. This was revealed in the present research study, with greater quality and quantity of school training being evident amongst PES than ECES, with the latter having attended a reduced number of conferences, seminars, and workshops. Moreover, training in innovative methodologies acquired by students with a teaching profile will assist their teaching practice in relation to educational environmental material. To this, we can also add the desire that one has to train themselves in an informal way.

For this, it is recommended to conduct a review of SDGs to implement educational actions within university life and study plans. This is especially the case for the specifics of education, which enables appropriate awareness to be acquired and professional skills to be developed within the working environment.

## Figures and Tables

**Table 1:** Categorisation system and coding.

Categories	Coding
Importance of SD in society	SD as an important problem for society
	SD as a factor to the progress of a country
Relevance of the ISRSD vs. Corporate Agents	ISRSD
	CSRSD of Social and Educational Agents
Attitudes and habits relative to SD	Pro-environmental attitudes
	Environmental care habits
Education for SD in schools: knowledge, attitudes and proposals	Knowledge of SD and its learning
	Attitudes towards ESD
	ESD proposals aimed at students

CSRSD: Corporate Social Responsibility for Sustainable Development, ESD: Education for Sustainable Development, ISRSD: Individual Social Responsibility for Sustainable Development, SD: Sustainable Development.

## Appendix A

**Table A1:** Interview for the opinions of the future teachers evaluated.

Questions
5. For a country to be considered as developed, which factors should we focus on: economic, social, work, educational, or environmental factors?
6. Following on from the previous question, number the options according to degree of importance and justify your response.
7. Do you consider consumption to be excessive?
8. Who is responsible for making the world more sustainable?
9. Who are those responsible: public administrations, businesses, individuals?
10. Do you think that we should strengthen education for consumers in classrooms?
11. As individuals, do you think that we have the power to resolve environmental problems?
12. Do you practice responsible consumption?
13. What do you know about the topic?
14. How would you promote responsible consumption amongst your students?

Note: The questions posed to the participants were formulated with the intention of responding to the information presented in the results, which corresponds to the categories presented in Table 1. However, the information collected in the different categories has been extracted from the data obtained in various questions, in accordance with participants’ speech. For instance, participants answered the first category using questions 5, 6, and 7; although some elements were also included in the speech related to questions 8 and 9. Yet, the questions were initially conceived to be answered specifically as follows, according to the code order: category 1 with questions 5, 6, and 7; category 2 with questions 8 and 9; category 3 with questions 11 and 12; and category 4 with questions 13, 10, and 14.

**Table A2:** Relationship between categories, codes, and frequency of quotes registered.

Categories	Coding	Frequency Early Childhood Education	Frequency Primary Education	Total Frequency
Importance of SD in society	SD as an important problem for society	44	37	81
	SD as a factor to the progress of a country	30	26	56
Relevance of the ISRSD vs. corporate agents	ISRSD of social and educational agents	53	49	102
	CSRSD of social and educational agents	31	31	62
Attitudes and habits relative to SD	Pro-environmental attitudes	65	69	134
	Environmental care habits	31	23	54
Education for SD in schools: knowledge, attitudes, and proposals	Knowledge of SD and its learning	18	11	29
	Attitudes towards ESD	63	39	102
	ESD proposals aimed at students	51	39	90
